# Crystal structure of poly[μ-aqua-bis(μ_3_-2-methylpropanoato-κ^4^ *O*:*O*,*O*′:*O*′)dipotassium]

**DOI:** 10.1107/S2056989020012591

**Published:** 2020-09-22

**Authors:** Jan Fábry, Erika Samolová

**Affiliations:** a Inst. of Physics of the Czech Academy of Sciences, Na Slovance 2, 182 21 Praha 8, Czech Republic

**Keywords:** methyl­propano­ate, metal–organic framework compounds, Cambridge Structural Database, crystal structure

## Abstract

The structure of the title compound, [K_2_(C_4_H_7_O_2_)_2_(H_2_O)]_*n*_, is composed of stacked sandwiches, which are formed by cation–oxygen bilayers surrounded by methyl­ethyl hydro­phobic chains. These sandwiches are held together by van der Waals inter­actions between the methyl­ethyl groups. The methyl­ethyl groups are disordered over two positions with occupancies 0.801 (3):0.199 (3). The potassium cations are coordinated by seven O atoms, which form an irregular polyhedron. There is a water mol­ecule, the oxygen atom of which is situated in a special position on a twofold axis (Wyckoff position 4*e*). The water H atoms are involved in O_water_—H⋯O_carbox­yl_ hydrogen bonds of moderate strength. These hydrogen bonds are situated within the cation–oxygen, *i.e.* hydro­philic, bilayer.

## Abstract 2

The structure of poly[bis­(μ_4_-2-methyl­propano­ato-η^2^-*O*-η^3^-*O*′)-μ_2_-aqua­dipotassium] (potassium isobutyrate hemihydrate) is reported. The structure is composed of cation–oxygen bilayers, which are surrounded by hydro­phobic methyl­ethyl chains on both sides. Stacking of these sandwiches forms the structure. The potassium cations are situated in an irregular coordination polyhedron composed of seven O atoms.

## Chemical context   

The structures of simple alkali 2-methyl­propano­ates (isobutyrates) have not been determined so far (as shown by a search of the Cambridge Structural Database, version 5.41, update of November 2019; Groom *et al.*, 2016 ▸) . In this context, ‘simple’ means a compound containing just one cationic species. The reason for this rather surprising fact may follow from the expected difficult crystallization (Mirnaya *et al.*, 1991 ▸). Moreover, the phases of isobutyrate salts are supposedly prone to undergo phase transitions due to the ordering of voluminous hydro­phobic methyl­ethyl chains by analogy to the phase transitions observed in alkali propionates and 2-methyl­propano­ates (Ferloni *et al.*, 1975 ▸).

The chemistry of water solutions and the corresponding solid phases of 2-methyl­propano­ates and other carboxyl­ates where the number of carbon atoms is greater than two differs from that of formates and acetates. The structures and chemistry of the former compounds are also affected by hydration (Mirnaya *et al.*, 1991 ▸). Hydration may take place because water mol­ecules compete with the carboxyl­ates in inclusion into the coordination sphere of the cations. Moreover, a rather tedious structure determination can be expected in alkanoates where the number of carbon atoms is greater than two because the organic chains tend to be positionally disordered and tend to exert large thermal agitation. This disorder, as well as the large displacement parameters, is related to the tendency to form different phases as pointed out above.

The structure determinations of 2-methyl­propano­ates as well as those of chemically related compounds with carboxyl­ates other than the formates and acetates show that their structures share the same tendency for the separation of metal cations, carboxyl­ate groups and sometimes water mol­ecules on the one hand from the organic chains on the other. The former groups are hydro­philic while the latter are hydro­phobic. The separation of these groups in these structures may be considered as an illustration of the alchemists’ experience expressed by the slogan *similis similibus solvuntur* on a microstructural level. This separation also refers to solvate mol­ecules and affects their orientation with regard to their hydro­philic and hydro­phobic ends. The compound *catena*-[tetra­kis­(μ_2_-isobutyrato-*O*,*O*,*O*′)bis­(isobutyrato-*O*,*O*′)tri­aqua­dicerium ethanol solvate] (refcode XALZAN; Malaestean *et al.*, 2012 ▸) can serve as an example.

Thus, the inter­molecular bonds in these structures can be divided into metal–oxygen bonds, O—H⋯O hydrogen bonds and van der Waals bonds between the hydro­phobic groups. The water mol­ecules as well as the solvate mol­ecules can be either coordinated to the cation or not while completing the hydro­philic part of the structures. At the same time, they are included into the hydrogen-bond pattern.

Correspondingly, the structures can be divided into the following classes (Table 1 ▸):

(i) Structures that are composed of clusters where the inner part is formed by hydro­philic parts while the outer skin is formed by hydro­phobic groups.

(ii) Structures that are formed by columns, the inter­ior of which is composed of the hydro­philic parts while the outer skin is hydro­phobic.

(iii) Layered structures that are composed of stacked sandwiches formed by cation–oxygen bilayers surrounded by hydro­phobic organic groups. These sandwiches are bonded by van der Waals forces.

In all of these structural types, water mol­ecules can occur; examples are given in Table 1 ▸.

So far, the structures of 2-methyl­propano­ates (isobutyrates) have been reviewed. The motif of stacked layers, however, seems to be typical for simple alkali alkanoates *M* ^+^C_*n*_H_2*n*+1_COO^−^, *n* > 2, as follows from the known structures of Li(C_3_H_5_O_2_) (refcodes OMERUV, OMERUV01 and OMERUV02; Martínez Casado *et al.*, 2009 ▸), and the recently determined series of structures of Na, K, Rb and Cs propano­ates (Fábry & Samolová, 2020 ▸), Tl(C_3_H_5_O_2_), *catena*-[(μ_2_-propano­ato)thallium(I)(propano­ato)thallium(I)] (WEWKAM; Martínez Casado *et al.*, 2010 ▸) and further from the structures of potassium acrylate and potassium methacrylate (refcodes VOVWOV and VOVWAH, respectively; Heyman *et al.*, 2020 ▸) as well as from the known structures with alkanoates with longer organic chains, *e.g.* potassium palmitate KC_16_H_31_O_2_ (KPALMA; Dumbleton & Lomer, 1965 ▸).

Thus, the typical motif of separated hydro­phobic and hydro­philic parts of the mol­ecules can be generalized for carboxyl­ates other than formates and acetates.

The physical properties of 2-methyl­propano­ates as well as other related carboxyl­ates C_*n*_H_2*n*+1_COO^−^, *n* > 2, hinder possible applications of these compounds, although there are some exceptions such as lanthanide zinc butyrates or their analogues, which have been applied for the synthesis of lanthanide–zinc–oxygen nanoparticles (Boyle *et al.*, 2010 ▸) or for gelation induced by ultrasound in presence of ZnO nanoparticles (Kotal *et al.*, 2010 ▸).
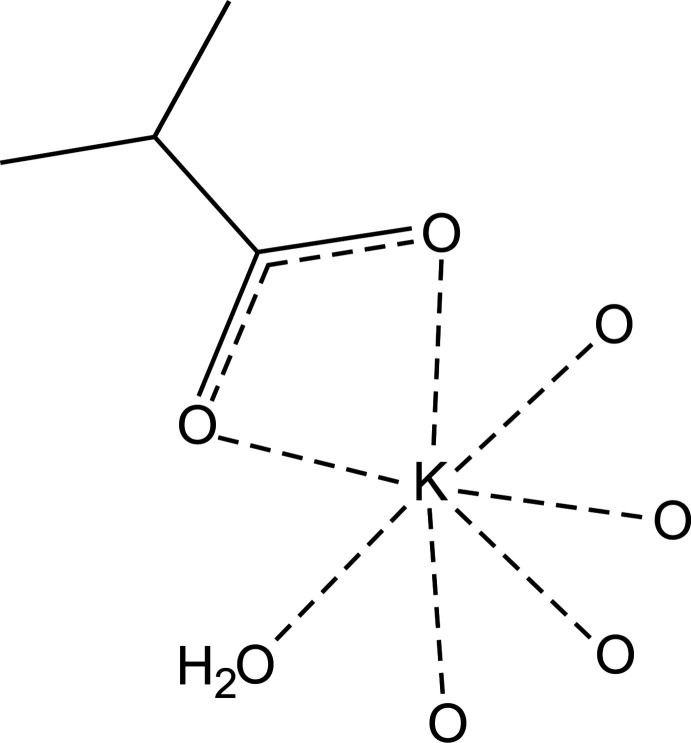


The aim of the present study was the preparation of potassium 2-methyl­propano­ate in order to fill the gap in the knowledge of these structures. Moreover, it was even more attractive to compare the sodium and potassium propano­ate structures (Fábry & Samolová, 2020 ▸) in which the methyl groups are situated in two positions related by rotation of 180° because such a positional disorder mimics the arrangement of both methyl­ethyl chains in 2-methyl­propano­ates by the demand for space. However, a crystal of a hydrated phase has been obtained, the structure of which is reported here. Still, the authors believe that this reported structure determination adds a piece of knowledge that could be helpful in understanding the structural features in simple alkali carboxyl­ates with C_*n*_H_2*n*+1_COO^−^, *n* > 2, and related structures.

## Structural commentary   

**Figure 1:**
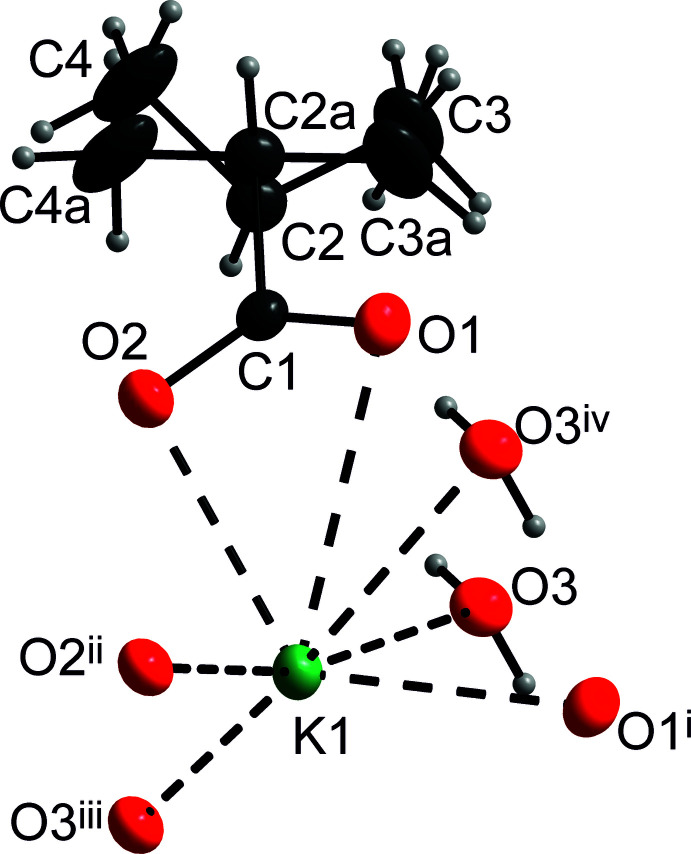
A view of the structural motif in the title compound (*DIAMOND*; Brandenburg, 2005 ▸): Displacement ellipsoids are shown at the 30% probability level. K, O, C and water H atoms are shown as green, red, black ellipsoids as well as gray spheres, respectively. Symmetry codes: (i) 1 − *x*, *y*, 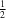 − *z*; (ii) 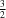 − *x*, −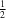 + *y*, 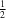 − *z*; (iii) 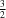 − *x*, 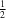 + *y*, 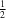 − *z*; (iv) *x*, −1 + *y*, *z*.

The structural unit of the title compound is shown in Fig. 1 ▸, which shows that the central cation is surrounded by seven oxygen atoms up to ∼3.33 Å. All of the oxygens stem from the carboxyl­ates except for the atom O3, which is a part of the coordinated water mol­ecule. The K—O bond distances are listed in Table 2 ▸. Five of them, *i.e.* O2, O2^ii^, O2^iii^, O3 and O3^iv^, form a tetra­gonal pyramid with O2 as its apex. Atoms O1 and O1^i^ complete the coordination polyhedron [symmetry codes: (i) 1 − *x*, *y*, 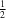 − *z*; (ii) 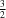 − *x*, −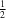 + *y*, 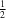 − *z*; (iii) 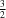 − *x*, 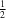 + *y*, 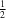 − *z*; (iv) *x*, −1 + *y*, *z*.) It is also worth mentioning that the distances between the cation and the oxygen atoms belonging to the same carboxyl­ate are quite different: K1—O1 = 3.1113 (13) Å and K1—O2 = 2.8056 (13) Å.

## Supra­molecular features   

**Figure 2:**
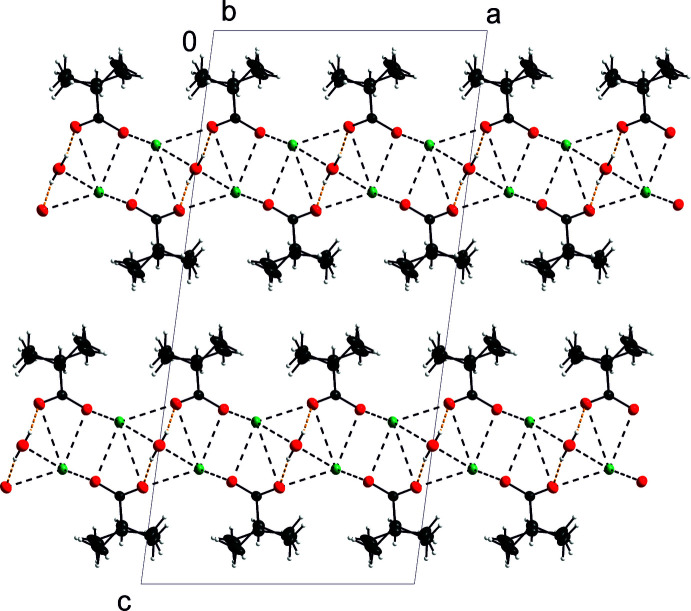
The packing of the mol­ecules in the title compound (*DIAMOND*; Brandenburg, 2005 ▸) viewed along the *b* axis. Displacement ellipsoids are shown at the 30% probability level. The colours are assigned to the atoms are as in Fig. 1 ▸.

The prominent feature of the title structure is the presence of an oxygen–metal bilayer, which is surrounded by methyl­ethyl chains on both sides (Fig. 2 ▸). This bilayer is composed of the cations and the oxygen atoms.

**Figure 3:**
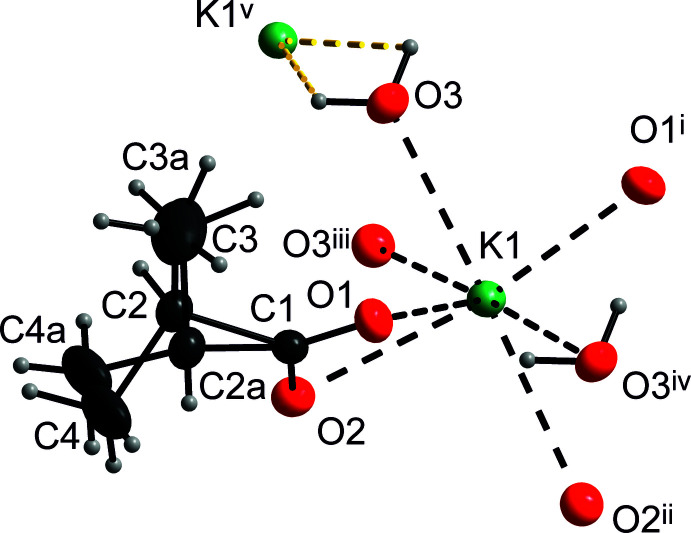
The structural motif showing the inter­action of a water mol­ecule to K1^v^. Displacement ellipsoids are shown at the 30% probability level. The colours are assigned to the atoms are as in Fig. 1 ▸. Symmetry codes as in Fig. 1 ▸ and (v) *x*, 1 + *y*, *z*.

Table 3 ▸ lists a pair of symmetry-equivalent O_water_—H⋯O_carboxyl­ate_ hydrogen bonds of moderate strength (Gilli & Gilli, 2009 ▸). These hydrogen bonds take place within the cation–oxygen bilayer (Fig. 2 ▸). Inter­estingly, the water hydrogen atoms, supposedly positively charged, are directed towards the more distant cation K1 [H1*O*3⋯K1^v^ and H1*O*3^i^⋯K1^v^ = 3.033 (19) and 3.01 (2) Å, respectively, see Fig. 3 ▸ and its caption]. This means that the positive-charge inter­action diminishes a cohesive weak inter­action O3⋯K1^v^, the bond valence of which is 0.0385 (1) (Brese & O’Keeffe, 1991 ▸). Other cohesive hydrogen-bonding inter­actions are listed in Table 3 ▸.

As stated above, methyl­ethyl chains surround the hydro­philic inner bilayer on both sides. The packing of these sandwiches forms the title structure. The sandwiches are held together by van der Waals forces. Table 4 ▸ lists these weak inter­actions. Their distances are about the same as those in dicalcium barium hexa­kis­(propano­ate) Ca_2_Ba(C_3_H_5_O_2_)_6_ [4.05 (2) Å; Stadnicka & Glazer, 1980 ▸] where disorder of the ethyl groups occurs. On the other hand, these inter­molecular distances are somewhat longer than in sodium and potassium propano­ates, where disorder of the ethyl groups has also been observed (Fábry & Samolová, 2020 ▸). In the latter structures, the distances between two ethyl groups while one of them is in a disordered position are as short as 2.609 (8) and 2.651 (9) Å, respectively, which is an indication of a dynamic disorder: *cf.* the discussion about the disorder in Ca_2_Ba(C_3_H_5_O_2_)_6_ by Stadnicka & Glazer (1980 ▸) according to whom the disorder is related to close C—C distances that are shorter than the sum of the van der Waals radii (about 4.5 Å). In the structurally related rubidium and caesium propano­ates, however, such an occupational disorder does not take place, most probably because of the longer distances between the ethyl groups in the latter structures. The shortest C—C distances in rubidium and caesium propano­ates are 3.908 (12) and 3.882 (13) Å, respectively.

## Synthesis and crystallization   

Preparation of potassium 2-methyl­propano­ate was intended. The compound was prepared by dissolving potassium carbonate sesquihydrate (1.50 g) with 2-methyl­propanoic acid (0.80 g) in the molar ratio 1:2 in water. The pH of the solution was adjusted to 6–7 by addition of several tenths of a ml of the acid.

The solution was filtered and the excess amount of water was evaporated at 313 K. Shortly before crystallization, a layer with a pronounced viscosity appeared on the surface of the solution. The crystals grew in the form of elongated colourless plates of several tenths of a mm in their longest direction.

## Structure determination and refinement   

**Figure 4:**
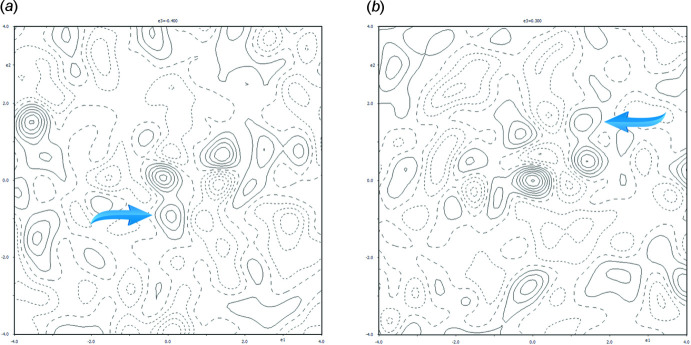
(*a*) The maximum (0.6025, 0.0146, 0.1014), which is indicated by the arrow, is in the vicinity of C2. The increment of positive (solid lines) and negative (dashed) contours are 0.1 e Å^−3^. The height of the indicated maximum is 0.33 e Å^−3^ in the depicted section. The structural model did not contain the atoms C2, C2*a*, C3, C3*a*, C4 and C4*a* and the attached H atoms. (*b*) The maximum (0.4677, 0.5409, 0.0940), which is indicated by the arrow, is in the vicinity of C3. The increment of positive (solid lines) and negative (dashed) contours are 0.1 e Å^−3^. The height of the maximum is 0.27 e Å^−3^ in the depicted section. The model did not contain the atoms C2, C2*a*, C3, C3*a*, C4 and C4*a* and the attached H atoms.

Crystal data, data collection and structure refinement details are summarized in Table 5 ▸. The refinement was carried out on the averaged set of independent diffractions. All of the non-hydrogen atoms were determined by *SHELXT* (Sheldrick, 2015 ▸). The structure was treated with consideration of a positional disorder of the methyl­ethyl chain. This disorder was revealed by a relatively high peak of residual electron density (0.68 e Å^−3^) in the vicinity of atom C2 (Fig. 4 ▸ *a*), which was pertinent to a model without assumed disorder. This residual peak was on the opposite side of the vector C2—H1*C*2 and was observable in the difference electron-density map using a model without the atoms C2, C3 and C4 as well as without the hydrogens attached to the latter carbons. This peak was assigned to a disordered atom C2 and denoted as C2*a*. Correspondingly, the carbon C3 was also disordered in the difference electron-density map (Fig. 4 ▸ *b*). Atoms C3 and C4 were split into the positions C3, C3*a* and C4, C4*a* after inclusion into the difference electron-density map.

The occupational parameters of these pairs of atoms, as well as of the attached hydrogens, were constrained so that their sum is equal to 1; the occupational parameter of atom C4 was refined. Each pair of these carbon atoms was constrained in such a way that the atom with the minor occupancy was assigned the same displacement parameters as the atom with the major occupancy. The carboxyl­ate carbon C1 was not split; the present model with the non-split carboxyl­ate carbon C1 was given preference because a splitting was too small and called for severe restraints of the C1—O1 and C1—O2 distances.

The methane­triyl hydrogens H1*c*2 and H1*c*2*a*, although observable, were placed in calculated positions. The latter hydrogens were refined under the following constraints: C_methane­tri­yl_—H_methane­tri­yl_ = 0.99 Å, *U*_iso_(H_methane­tri­yl_) = 1.2*U*_eq_(C_methane­tri­yl_). Subsequently, after the anisotropic refinement of the non-hydrogen atoms with the methane­triyl hydrogen, the difference electron-density map revealed the methyl hydrogens. All of the methyl hydrogens were discernible in the difference electron-density maps. The hydrogens belonging to the major disorder component were found at first and then, after the refinement had converged, the other methyl hydrogens were found and refined. The methyl hydrogens were refined under the following constraints: C_meth­yl_—H_meth­yl_ = 0.96 Å, *U*_iso_(H_methyl_) = 1.5*U*_eq_(C_methyl_). A following difference electron-density map revealed the water hydrogen, which was situated in a general position in contrast to its carrier O3. The water hydrogen was refined using the angle restraint H1*O*3—O3—H1*O*3^i^ [symmetry code: (i) 1 − *x*, *y*, 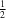 − *z*] = 105.00 (1)° while *U*_iso_(H1*O*3) = 1.5*U*_eq_(O3). A trial refinement showed that the water oxygen was fully occupied. The C1—C2, C1—C2*a* bonds were restrained to be equal [1.540 (1) Å] as were C2—C3, C2—C3*a* and C2—C4, C2—C4*a* [1.500 (1) Å]. These values were found to yield the lowest *R* factors. Moreover, angle restraints to C3—C2—C4 and C3*a*—C2*a*—C4*a* were also applied. Of course, these C—C distances are affected by a large thermal agitation and are less reliable, as are the geometric parameters, compared to those of atom C1.

## Supplementary Material

Crystal structure: contains datablock(s) global, I. DOI: 10.1107/S2056989020012591/dj2009sup1.cif

Structure factors: contains datablock(s) I. DOI: 10.1107/S2056989020012591/dj2009Isup2.hkl

Supporting information file. DOI: 10.1107/S2056989020012591/dj2009Isup3.smi

CCDC reference: 2032320

Additional supporting information:  crystallographic information; 3D view; checkCIF report

## Figures and Tables

**Table 1:** Overview of structural types observed in isobutyrates

Compound	Refcode	Reference
Water-free clusters		
1	OHOXUF	Malaestean *et al.* (2009 ▸)
2	GEWFUL	Malaestean *et al.* (2013*a* ▸)
3	NAGQUI	Coker *et al.* (2004 ▸)
		
Clusters inter­connected by water mol­ecules		
4	NAGQUI	Coker *et al.* (2004 ▸)
		
Water-free columns		
5	PENJUN	Ilina *et al.* (1992 ▸)
6	TAHXOR	Boyle *et al.* (2010 ▸)
7	TAHXOR01	Bierke & Meyer (2008 ▸)
8	TAHXOR02	Kotal *et al.* (2010 ▸)
		
Columns inter­connected by water mol­ecules		
9	MECVAU	Skelton & Deacon (2017 ▸)
10	XALZAN	Malaestean *et al.* (2012 ▸)
		
Water-free layered structures		
11	KELKOE	Skelton & Deacon (2017 ▸)
12	LUHGOK	Yuranov & Dunaeva (1989 ▸)
		
Structures with layers inter­connected by water mol­ecules		
13	VIQTOG	Malaestean *et al.* (2013*b* ▸)
14	POSCIJ	Troyanov *et al.* (1993 ▸)
15	SAJMUO	Fischer *et al.* (2017 ▸)

Compound names, 1: bis(μ_4_-oxo)dodeca­kis­(μ_3_-isobutyrato)hexa­kis­(μ_2_-isobutyrato)bis(isobutyric acid)-bis­(propanol)-octa-manganese(II)-di-manganese(III) dihydrate; 2: hexa­kis­(μ_3_-isobutyrato)hexa­kis­(μ_2_-isobutyrato)hexa­kis­(2-methyl­propanoic acid)hexa­man­gan­ese; 3: hexa­kis­[bis­(μ_2_-2-methyl­propano­ato)(2-methyl­propanoic acid)magnesium]; 4: bis­(μ_4_-oxo)dodeca­kis­(μ_3_-isobutyrato)hexa­kis­(μ_2_-isobutyrato)bis­(isobutyric acid)bis­(propanol)octa­manganese(II)dimanganese(III) propanol solvate; 5: bis­[(μ_3_-isobutyrylato-*O*,*O*′)(μ_2_-isobutyrylato)copper(II)]; 6: *catena*-[tetra­kis­(μ_2_-2-methyl­prop­ano­ato)dizinc]; 7: *catena*-[tetra­kis­(μ_2_-2-methyl­propano­ato)dizinc]; 8: *catena*-[tetra­kis­(μ_2_-2-methyl­propano­ato)dizinc); 9: *catena*-[tetra­kis­(μ-2-methyl­propano­ato)bis­(2-methyl­propano­ato)tri­aqua­dilanthanum(III) hydrate; 10: *catena*-[tetra­kis­(μ_2_-isobutyrato-*O*,*O*,*O*′)bis­(isobutyrato-*O*,*O*′)tri­aqua­dicerium ethanol solvate]; 11: *catena*-[hexa­kis­(μ-methyl­propano­ato)dilutetium]; 12: di­aqua­bis­(isobutyrato)dioxouranium(VI); 13: *catena*-[(μ_2_-2-methyl­propano­ato)(2-methyl­propano­ato)tri­aqua­magnesium monohydrate]; 14: *catena*-[hexa­kis­(μ_2_-isobutyrato)aqua­dierbium monohydrate]; 15: *catena*-[bis­(μ-2-meth­yl­propano­ato)(μ-aqua)­cobalt(II) monohydrate].

**Table 2:** Selected bond lengths (Å)

K1—O1	3.1113 (13)	K1—O2^iii^	2.7330 (12)
K1—O1^i^	2.6951 (14)	K1—O3^iv^	3.3351 (13)
K1—O2	2.8056 (13)	K1—O3	2.7693 (12)
K1—O2^ii^	2.7360 (12)		

Symmetry codes: (i) 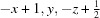; (ii) 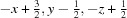; (iii) 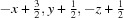; (iv) 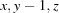.

**Table 3:** Hydrogen-bond geometry (Å, °)

*D*—H⋯*A*	*D*—H	H⋯*A*	*D*⋯*A*	*D*—H⋯*A*
O3—H1O3⋯O1^v^	0.83 (2)	1.92 (2)	2.7358 (17)	167 (2)
O3—H1O3^i^⋯O1^vi^	0.83 (2)	1.92 (2)	2.7358 (17)	167 (2)

Symmetry codes: (i) 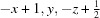; (v) 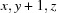; (vi) 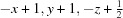.

**Table 4:** C_methyl­ene_—C_meth­yl_ and C_meth­yl_—C_meth­yl_ inter­molecular distances (Å) in the title structure of up to 4.5 Å C2, C2*a* correspond to the methyl­ene carbon atoms while C3, C3*a* and C4, C4*a* correspond to methyl atoms.

C2⋯C4^v^	4.251 (6)	C3⋯C4^vi^	3.992 (6)
C3⋯C4^viii^	4.136 (6)	C4⋯C4^ix^	3.984 (5)
C3*a*⋯C4*a* ^vii^	4.17 (2)	C3*a*⋯C4*a* ^vi^	4.25 (2)
C4*a*⋯C4*a* ^ix^	4.31 (2)	C2*a*⋯C3*a* ^*x*^	3.97 (2)
C2*a*⋯C4*a* ^*x*^	3.884 (19)	C2⋯C2*a* ^v^	3.960 (10)
C2⋯C4*a* ^*x*^	4.479 (16)	C3⋯C2*a* ^v^	4.128 (11)
C3⋯C3*a* ^*x*^	4.44 (2)	C3⋯C4*a* ^vii^	4.000 (15)
C3⋯C4*a* ^vi^	4.207 (15)	C4⋯C3*a* ^*x*^	4.48 (2)
C4⋯C3*a* ^xi^	4.087 (14)	C4⋯C3*a* ^viii^	4.43 (3)
C4⋯C4*a* ^*x*^	3.767 (17)	C4⋯C4*a* ^ix^	4.051 (17)

Symmetry codes: (v) *x*, *y* + 1, *z*; (vi) *x* − 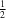, *y* + 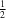, *z*; (vii) *x* − 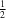, *y* − 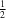, *z*; (viii) −*x* + 1, −*y*, −*z*; (ix) −*x* + 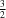, −*y* + 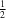, −*z*; (*x*) *x*, *y* − 1, *z*; (xi) *x* + 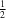, *y* − 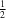, *z*; (xii) *x* + 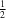, *y* + 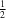, *z*.

**Table 5:** Experimental details

Crystal data	
Chemical formula	[K_2_(C_4_H_7_O_2_)_2_(H_2_O)]
*M*_r_	270.4
Crystal system, space group	Monoclinic, *C*2/*c*
Temperature (K)	240
*a*, *b*, *c* (Å)	11.9190 (5), 4.5454 (2), 24.3172 (9)
β (°)	97.517 (1)
*V* (Å^3^)	1306.10 (9)
*Z*	4
Radiation type	Cu *K*α
μ (mm^−1^)	6.45
Crystal size (mm)	0.34 × 0.14 × 0.04

Data collection	
Diffractometer	Bruker D8 VENTURE Kappa Duo PHOTON 100 CMOS
Absorption correction	Multi-scan (*SADABS*; Bruker, 2017 ▸)
*T*_min_, *T*_max_	0.219, 0.765
No. of measured, independent and observed [*I* > 3σ(*I*)] reflections	10532, 1269, 1212
*R*_int_	0.036
(sin θ/λ)_max_ (Å^−1^)	0.618

Refinement	
*R*[*F* > 3σ(*F*)], *wR*(*F*), *S*	0.028, 0.082, 2.48
No. of reflections	1269
No. of parameters	82
No. of restraints	9
H-atom treatment	H atoms treated by a mixture of independent and constrained refinement
Δρ_max_, Δρ_min_ (e Å^−3^)	0.19, −0.22

Computer programs: *Instrument Service* and *SAINT* (Bruker, 2017 ▸), *SHELXT* (Sheldrick, 2015 ▸), *JANA2006* (Petříček *et al.*, 2014 ▸) and *DIAMOND* (Brandenburg, 2015).

## supplementary crystallographic information

### Crystal data

**Table:**

[K_2_(C_4_H_7_O_2_)_2_(H_2_O)]	*F*(000) = 568
*M**_r_* = 270.4	There have been used diffractions with I/σ(I)>20 for the unit cell determination.
Monoclinic, *C*2/*c*	*D*_x_ = 1.375 Mg m^−^^3^
Hall symbol: -C 2yc	Cu *K*α radiation, λ = 1.54178 Å
*a* = 11.9190 (5) Å	Cell parameters from 8438 reflections
*b* = 4.5454 (2) Å	θ = 7.4–72.2°
*c* = 24.3172 (9) Å	µ = 6.45 mm^−^^1^
β = 97.517 (1)°	*T* = 240 K
*V* = 1306.10 (9) Å^3^	Plate, colourless
*Z* = 4	0.34 × 0.14 × 0.04 mm

### Data collection

**Table:**

Bruker D8 VENTURE Kappa Duo PHOTON 100 CMOS diffractometer	1269 independent reflections
Radiation source: IµS micro-focus sealed tube	1212 reflections with *I* > 3σ(*I*)
Helios Cu multilayer optic monochromator	*R*_int_ = 0.036
φ and ω scans	θ_max_ = 72.2°, θ_min_ = 7.4°
Absorption correction: multi-scan (*SADABS*; Bruker, 2017)	*h* = −14→14
*T*_min_ = 0.219, *T*_max_ = 0.765	*k* = −5→5
10532 measured reflections	*l* = −30→30

### Refinement

**Table:**

Refinement on *F*^2^	94 constraints
*R*[*F* > 3σ(*F*)] = 0.028	Primary atom site location: dual
*wR*(*F*) = 0.082	H atoms treated by a mixture of independent and constrained refinement
*S* = 2.48	Weighting scheme based on measured s.u.'s *w* = 1/(σ^2^(*I*) + 0.0004*I*^2^)
1269 reflections	(Δ/σ)_max_ = 0.035
82 parameters	Δρ_max_ = 0.19 e Å^−^^3^
9 restraints	Δρ_min_ = −0.22 e Å^−^^3^

### Special details

**Table:**

Refinement. The reflections 2 2 0, 4 2 0, 5 1 7 and 3 3 0 were excluded from the refinement because |I_obs_-I_calc_|>15σ(I_obs_).

### Fractional atomic coordinates and isotropic or equivalent isotropic displacement parameters (Å^2^)

**Table:**

	*x*	*y*	*z*	*U*_iso_*/*U*_eq_	Occ. (<1)
K1	0.65739 (3)	0.08834 (7)	0.292673 (13)	0.03769 (13)	
O1	0.54092 (11)	−0.0749 (2)	0.17459 (5)	0.0474 (4)	
O2	0.71739 (10)	0.0879 (2)	0.18489 (5)	0.0451 (4)	
O3	0.5	0.5048 (4)	0.25	0.0442 (5)	
H1o3	0.521 (2)	0.616 (3)	0.2262 (8)	0.0664*	
C1	0.62018 (14)	0.0693 (3)	0.15869 (6)	0.0357 (4)	
C2	0.59779 (18)	0.2545 (5)	0.10542 (7)	0.0445 (6)	0.801 (3)
H1c2	0.62015	0.458674	0.115969	0.0533*	0.801 (3)
C2a	0.5957 (9)	0.124 (2)	0.09573 (13)	0.0445 (6)	0.199 (3)
H1c2a	0.590888	−0.055688	0.072613	0.0533*	0.199 (3)
C3	0.4752 (2)	0.2726 (12)	0.0811 (2)	0.0828 (14)	0.801 (3)
H1c3	0.433861	0.380314	0.105886	0.1242*	0.801 (3)
H2c3	0.468892	0.371338	0.045975	0.1242*	0.801 (3)
H3c3	0.444536	0.077688	0.076086	0.1242*	0.801 (3)
C3a	0.4876 (10)	0.297 (4)	0.0912 (13)	0.0828 (14)	0.199 (3)
H1c3a	0.442774	0.254928	0.056368	0.1242*	0.199 (3)
H2c3a	0.44613	0.243332	0.120994	0.1242*	0.199 (3)
H3c3a	0.504672	0.50346	0.093401	0.1242*	0.199 (3)
C4	0.6678 (4)	0.1371 (12)	0.06307 (12)	0.1008 (19)	0.801 (3)
H1c4	0.743884	0.103888	0.080315	0.1512*	0.801 (3)
H2c4	0.635891	−0.044833	0.048302	0.1512*	0.801 (3)
H3c4	0.668259	0.277338	0.033579	0.1512*	0.801 (3)
C4a	0.6882 (11)	0.314 (4)	0.0786 (7)	0.1008 (19)	0.199 (3)
H1c4a	0.669733	0.367954	0.040336	0.1512*	0.199 (3)
H2c4a	0.695838	0.487804	0.101114	0.1512*	0.199 (3)
H3c4a	0.758165	0.20639	0.083389	0.1512*	0.199 (3)

### Atomic displacement parameters (Å^2^)

**Table:**

	*U*^11^	*U*^22^	*U*^33^	*U*^12^	*U*^13^	*U*^23^
K1	0.0335 (2)	0.0384 (2)	0.0413 (2)	0.00132 (11)	0.00537 (14)	0.00039 (11)
O1	0.0463 (7)	0.0488 (7)	0.0492 (7)	−0.0052 (5)	0.0138 (5)	0.0043 (5)
O2	0.0387 (6)	0.0496 (6)	0.0457 (7)	0.0038 (5)	0.0002 (5)	0.0011 (4)
O3	0.0510 (10)	0.0318 (7)	0.0490 (9)	0	0.0031 (7)	0
C1	0.0375 (8)	0.0359 (7)	0.0345 (8)	0.0047 (6)	0.0075 (6)	−0.0002 (5)
C2	0.0494 (10)	0.0422 (12)	0.0417 (10)	0.0012 (11)	0.0056 (8)	0.0081 (9)
C2a	0.0494 (10)	0.0422 (12)	0.0417 (10)	0.0012 (11)	0.0056 (8)	0.0081 (9)
C3	0.0537 (14)	0.127 (3)	0.061 (3)	0.0035 (16)	−0.0173 (15)	0.0378 (19)
C3a	0.0537 (14)	0.127 (3)	0.061 (3)	0.0035 (16)	−0.0173 (15)	0.0378 (19)
C4	0.109 (3)	0.155 (5)	0.0460 (19)	0.053 (3)	0.038 (2)	0.029 (2)
C4a	0.109 (3)	0.155 (5)	0.0460 (19)	0.053 (3)	0.038 (2)	0.029 (2)

### Geometric parameters (Å, º)

**Table:**

K1—O1	3.1113 (13)	C2—C4	1.505 (4)
K1—O1^i^	2.6951 (14)	C2a—H1c2a	0.99
K1—O2	2.8056 (13)	C2a—C4a	1.500 (18)
K1—O2^ii^	2.7360 (12)	C3—H1c3	0.96
K1—O2^iii^	2.7330 (12)	C3—H2c3	0.96
K1—O3^iv^	3.3351 (13)	C3—H3c3	0.96
K1—O3	2.7693 (12)	C3a—H1c3a	0.96
O1—C1	1.252 (2)	C3a—H2c3a	0.96
O2—C1	1.2499 (19)	C3a—H3c3a	0.96
O3—H1o3	0.83 (2)	C4—H1c4	0.96
O3—H1o3^i^	0.83 (2)	C4—H2c4	0.96
C1—C2	1.539 (2)	C4—H3c4	0.96
C1—C2a	1.541 (4)	C4a—H1c4a	0.96
C2—C3	1.505 (4)	C4a—H2c4a	0.96
C2—C3a	1.328 (13)	C4a—H3c4a	0.96
			
O1—K1—O1^i^	84.51 (4)	O2—C1—C2a	122.5 (4)
O1—K1—O2	43.54 (3)	C1—C2—H1c2	106.47
O1—K1—O2^ii^	98.72 (3)	C1—C2—C3	114.4 (2)
O1—K1—O2^iii^	123.27 (4)	C1—C2—C4	109.3 (2)
O1—K1—O3^iv^	50.08 (2)	H1c2—C2—C3	105.38
O1—K1—O3	67.58 (3)	H1c2—C2—C4	110.8
O1^i^—K1—O2	128.05 (4)	C3—C2—C4	110.4 (3)
O1^i^—K1—O2^ii^	100.88 (4)	H1c2a—C2a—C3a	113.92
O1^i^—K1—O2^iii^	130.31 (4)	H1c2a—C2a—C4a	107.85
O1^i^—K1—O3^iv^	52.67 (3)	C3a—C2a—C4a	109.5 (12)
O1^i^—K1—O3	73.84 (3)	C2—C3—H1c3	109.47
O2—K1—O2^ii^	89.24 (4)	C2—C3—H2c3	109.47
O2—K1—O2^iii^	89.30 (4)	C2—C3—H3c3	109.47
O2—K1—O3^iv^	84.87 (2)	H1c3—C3—H2c3	109.47
O2—K1—O3	83.88 (2)	H1c3—C3—H3c3	109.47
O2^ii^—K1—O2^iii^	112.43 (4)	H2c3—C3—H3c3	109.47
O2^ii^—K1—O3^iv^	70.78 (3)	C2a—C3a—H1c3a	109.47
O2^ii^—K1—O3	165.48 (4)	C2a—C3a—H2c3a	109.47
O2^iii^—K1—O3^iv^	173.34 (3)	C2a—C3a—H3c3a	109.47
O2^iii^—K1—O3	80.35 (4)	H1c3a—C3a—H2c3a	109.47
O3^iv^—K1—O3	95.81 (3)	H1c3a—C3a—H3c3a	109.47
K1—O1—K1^i^	87.96 (4)	H2c3a—C3a—H3c3a	109.47
K1—O2—K1^ii^	90.76 (4)	C2—C4—H1c4	109.47
K1—O2—K1^iii^	90.70 (4)	C2—C4—H2c4	109.47
K1^ii^—O2—K1^iii^	112.43 (4)	C2—C4—H3c4	109.47
K1—O3—K1^v^	95.806 (12)	H1c4—C4—H2c4	109.47
K1—O3—K1^i^	93.77 (5)	H1c4—C4—H3c4	109.47
K1—O3—K1^vi^	170.43 (4)	H2c4—C4—H3c4	109.47
K1^v^—O3—K1^i^	170.43 (4)	H1c2—C4a—C2a	66.18
K1^v^—O3—K1^vi^	74.62 (3)	C2a—C4a—H1c4a	109.47
K1^i^—O3—K1^vi^	95.806 (12)	C2a—C4a—H2c4a	109.47
H1o3—O3—H1o3^i^	105.0 (16)	C2a—C4a—H3c4a	109.47
O1—C1—O2	124.40 (13)	H1c4a—C4a—H2c4a	109.47
O1—C1—C2	119.45 (14)	H1c4a—C4a—H3c4a	109.47
O1—C1—C2a	109.8 (4)	H2c4a—C4a—H3c4a	109.47
O2—C1—C2	116.04 (14)		

Symmetry codes: (i) −*x*+1, *y*, −*z*+1/2; (ii) −*x*+3/2, *y*−1/2, −*z*+1/2; (iii) −*x*+3/2, *y*+1/2, −*z*+1/2; (iv) *x*, *y*−1, *z*; (v) *x*, *y*+1, *z*; (vi) −*x*+1, *y*+1, −*z*+1/2.

### Hydrogen-bond geometry (Å, º)

**Table:**

*D*—H···*A*	*D*—H	H···*A*	*D*···*A*	*D*—H···*A*
O3—H1O3···O1^v^	0.83 (2)	1.92 (2)	2.7358 (17)	167 (2)
O3—H1O3^i^···O1^vi^	0.83 (2)	1.92 (2)	2.7358 (17)	167 (2)

Symmetry codes: (i) −*x*+1, *y*, −*z*+1/2; (v) *x*, *y*+1, *z*; (vi) −*x*+1, *y*+1, −*z*+1/2.
